# Synthesis and Characterization of Novel Pyridine Periodic Mesoporous Organosilicas and Its Catalytic Activity in the Knoevenagel Condensation Reaction

**DOI:** 10.3390/ma13051097

**Published:** 2020-03-02

**Authors:** Fatemeh Rajabi, Arezoo Zare Ebrahimi, Ahmad Rabiee, Antonio Pineda, Rafael Luque

**Affiliations:** 1 Department of Science, Payame Noor University, P.O. Box: 19395-4697, Tehran 19569, Iran; 2 Iran Polymer and Petrochemical Institute (IPPI), P.O. Box 112/14975, Tehran 19569, Iran; a.rabbii@ippi.ac.ir; 3 Department of Organic Chemistry, University of Cordoba, Ed. Marie Curie (C 3), Campus of Rabanales, Ctra Nnal IV-A, Km 396, E14014 Cordoba, Spain; q82pipia@uco.es (A.P.); q62alsor@uco.es (R.L.)

**Keywords:** periodic mesoporous organosilica, pyridine carboxamide, base catalysts, Knoevenagel condensation

## Abstract

The preparation of novel organic-inorganic hybrid mesoporous organosilica containing pyridinedicarboxamide functional groups uniformly distributed inside the nanostructured pore walls has been addressed. The mesoporosity and uniformity of the synthesized nanomaterials were characterized by different techniques such as nitrogen adsorption/desorption measurements and powder X-ray diffraction (PXRD). Additionally, the presence of the pyridinedicarboxamide in the pore walls of the nanomaterials was assessed by Fourier-transform infrared spectroscopy (FT-IR), as well as ^29^Si and ^13^C solid-state cross-polarization and magic angle spinning nuclear magnetic resonance (CP/MAS-NMR). The Knoevenagel condensation of aldehydes with active methylene compounds was carried out over the pyridinedicarboxamide functionalized mesoporous organosilica, which has been proven to be an efficient heterogeneous basic catalyst in the presence of ethanol as solvent. The catalytic activity of the investigated materials was investigated in the Knoevenagel condensation between malononitrile and several benzaldehyde derivatives exhibiting a high conversion (>90%) and excellent selectivity toward the final condensation products under very mild reaction conditions. Furthermore, the catalyst stability is noteworthy as it could be recycled and reused at least twelve times without any significant change in the performance.

## 1. Introduction

The design of mesoporous functionalized silicate catalysts has been targeted since the development of the M41S silicate materials. Many alternatives have been proposed for the introduction of functionalities in mesoporous silicates. Among such options to load active sites on inert silicates, these can be classified as direct or “in situ” methodologies and post-synthetic approaches [1]. Among these last strategies, grafting [2], wet impregnation [3], or other most recently reported approaches such as ball-milling [4] can be included. Alternatively, the “in situ” methodologies go through the addition of the functional group directly into the sol–gel mixture. Thus, silicates can be modified “in situ” with metals, including zirconium and aluminum, among others, as well as organic functionalities. In this sense, periodic mesoporous organosilicas (PMOs) represent a class of hybrid nanomaterials with the presence of organic bridging groups incorporated into the highly ordered silica frameworks via covalent Si-C bonds [5,6,7]. These materials are very attractive because of the combination of a stable inorganic support together with the possibility of material functionalization with a wide range of organic moieties homogeneously distributed among the solid structure. PMOs, owing to the possibility of incorporating different functionalities into their walls, may have a potential use in a wide variety of applications such as catalysis [8,9,10], gas adsorption [11,12], decontamination [13], optical purposes [14], microelectronics [15], and drug delivery [16].

The common preparation method for PMO materials involves hydrolysis and the subsequent polycondensation of polysilsesquioxane, a bis-silylated organosilica precursor, around a template. After aging and formation of the organosilica, the template is removed and a porous structure of PMO material is obtained. Although the bridge of the silane is a key factor, there are many other factors that can affect PMO preparation including template, swelling agents, additives, catalyst, pH, temperature, reaction time, and stirring rate [6]. Various classical organic bridging groups have been widely used including very simple units such as methane, ethane, ethene, benzene, and so on, in addition to alternative organic moieties, such as bridging molecules containing heterocyclic and chiral molecules. The organic function in the silica bridge will determine the chemical and physical properties of the PMO material such as a hydrophobic or hydrophilic surface, hydrothermal and mechanical stability, chemical reactivity, and the potential use of the materials.

Regarding catalytic applications, the number of reactions catalyzed by acidic PMOs reported is much higher than the number of reactions assisted by the basic PMOs catalysts. For instance, a basic urea-derived framework was used as the catalyst for CO_2_ coupling with epoxides [17]. Further, the use of amine bridges PMO materials has been reported as a catalyst for several reactions catalyzed by basic sites such as the Knoevenagel reaction or the solvent-free Henry reaction [18]. More recently, imine bridged PMOs were synthesized and successfully employed in the Knoevenagel condensation between aldehydes and ethyl cyanoacetate in water [19].

In this work, we report the incorporation of a pyridine dicarboxylate as bridging groups uniformly distributed into the silica framework as basic PMO material, developing in this way a crystal-like PMO catalyst containing heterocyclic pyridine dicarboxylate groups from a newly designed 2,6-pyridinedicarboxamide triethoxysilane precursor in the presence of a nonionic surfactant template. Such pyridinedicarboxamide units aim to be densely and regularly incorporated with in the highly ordered structure of the pore walls. The catalytic performance of as-synthesised materials was investigated in the Knoevenagel condensation reaction between aromatic aldehydes and malononitrile using ethanol as a solvent.

## 2. Results and Discussion

**Scheme 1:**
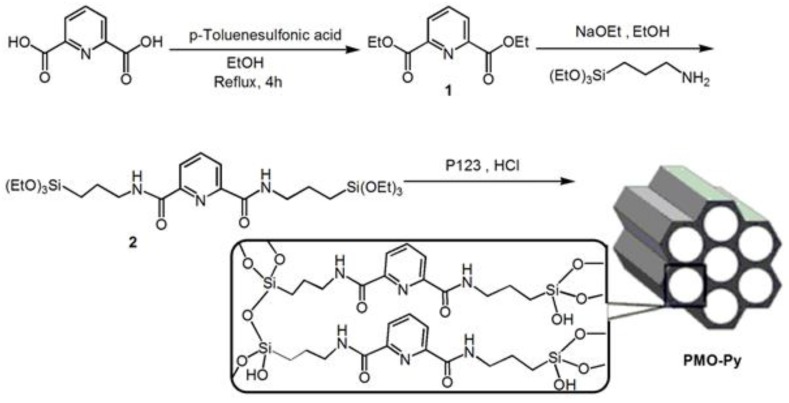
Illustrative representation of the synthesis of pyridine loaded periodic mesoporous organosilica (PMO) materials.

Herein, novel PMO material bearing pyridinedicarboxamide bridging groups (PMO-Py), bis(3-(triethoxysilyl)propyl)pyridine-2,6-dicarboxamide (2), were synthesized according to a two-step approach described on Scheme 1. Firstly, the formation of diethyl pyridine-2,6-dicarboxylate takes place via an acid-catalyzed esterification of pyridine-2,6-dicarboxylic acid with ethanol. Subsequently, the ester (1) formed in the first step undergoes an amidation reaction catalyzed by sodium ethoxide using 3-(triethoxysilyl)propyl amine, as outlined in Scheme 1. Eventually, the PMO-Py material was then synthesized by hydrolysis and condensation of in aqueous acidic solution in the presence of nonionic triblock copolymer surfactant P123 as a structure directing agent (SDA).

**Figure 1:**
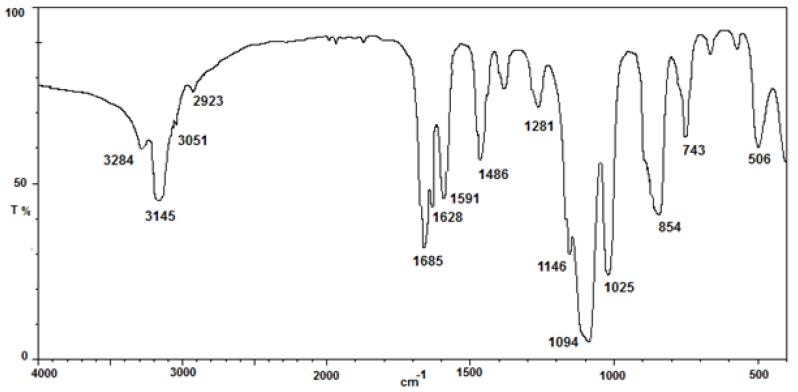
Fourier-transform infrared spectroscopy (FT-IR) spectrum for the periodic mesoporous organosilica (PMO) material bearing pyridinedicarboxamide bridging groups.

The efficient incorporation of pyridinedicarboxamide moieties into the material structure was confirmed by Fourier-transform infrared spectroscopy (FT-IR), which is depicted on Figure 1. In this sense, the presence of both silica framework as well as the organic moieties was confirmed. Firstly, the characteristic bands at 1628 cm^−^1 and 1595 cm^−1^ corresponding to the C=N stretching vibration and C=C stretching vibration of the pyridine ring, respectively, can be observed. In addition, the carbonyl group (C=O) stretching vibration band was detected at 1685 cm^−1^, which may confirm the presence of the amide in the as-synthesised material. Furthermore, the amide occurrence in the material can be confirmed by the presence of the N-H stretching vibration band (3284 cm^−1^) together with the presence at ~3051 cm^−1^ and 2923 cm^−1^ can be attributed to asymmetric and symmetric C–H stretching corresponding to the pyridine ring and the propyl chain, respectively. In addition, other bands observable on Figure 1 are the C–C stretching vibration, which is found at 1486 cm^−1^; C–C, C–O; and C–H bending vibrations, which were found in the region between 500 and 1000 cm^−1^. Regarding to silica framework, the characteristic stretching vibrations of Si–O–Si frameworks at approximately 506, 743, and 1096 cm^−1^ can be clearly observed. The condensed silica network was reported to exhibit a wide absorption band at about 3145 cm^−1^, attributed to the –OH stretching vibrations of silanol groups. These results point out that the pyridinedicarboxamide group was successfully incorporated into the PMO framework.

**Figure 2:**
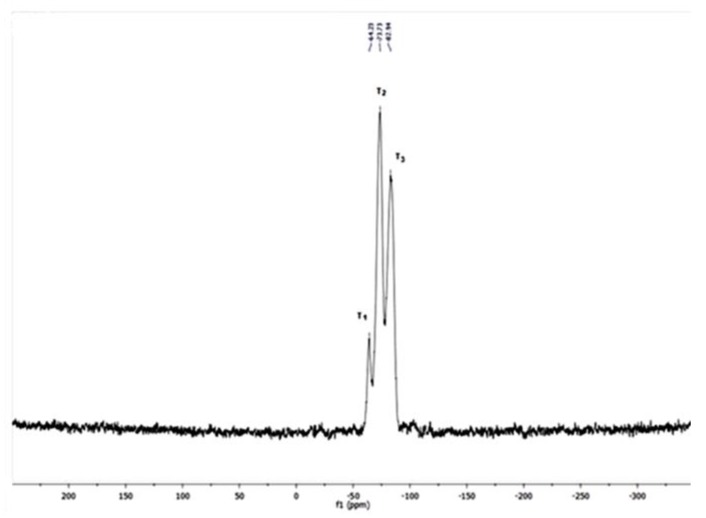
^29^Si CP/MAS nuclear magnetic resonance (NMR) spectrum of the pyridine periodic mesoporous organosilica (PMO-Py).

Besides FT-IR analysis, solid-state ^29^Si and ^13^C nuclear magnetic resonance (NMR) (Figure 2) measurements were performed to obtain additional structural information and confirm the incorporation of pyridinedicarboxamide bridging groups in the material. Regarding the solid-state ^29^Si NMR spectrum, there are signals with high intensity attributed to T1 species (CSi(OSi)(OH)_2_) at 66 ppm, T2-silicon species (CSi(OH)(OSi)_2_) at −70.8 ppm, and T3 species (CSi(OSi)_3_) at −78.8 ppm [20]. Then, it can be said that Si atoms are bound to an organic group and three O atoms(R–SiO_3_), which agrees with the expected structure in the material. Furthermore, the absence of any Q signals, which is because of the Si atoms linked to four O atoms [21], points out that the Si–C bonds remain intact during the functionalization processes.

**Figure 3:**
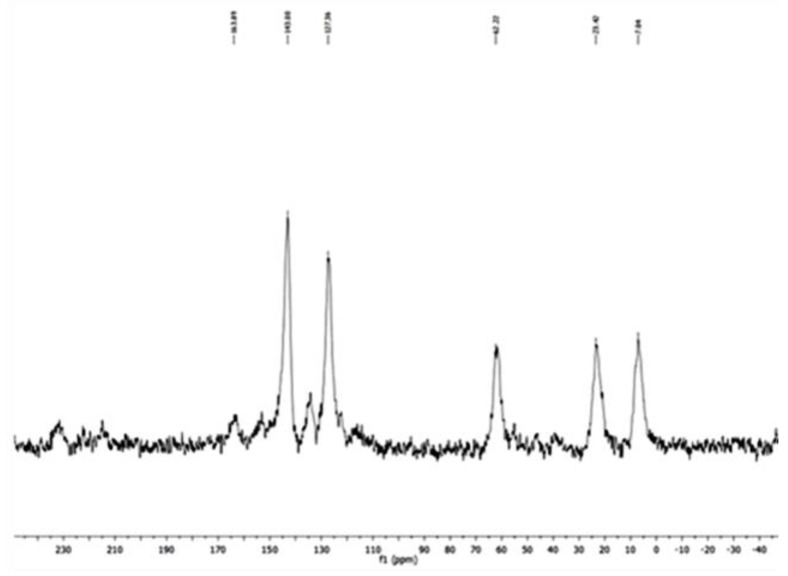
^13^C CP/MAS NMR spectrum of the PMO-Py material bearing pyridinedicarboxamide.

^13^C cross-polarization and magic angle spinning (CP/MAS) NMR (Figure 3) measurements confirmed the successful incorporation of pyridinecarboxamide group into the PMO framework. The ^13^C CP/MAS NMR spectrum of the pyridine ring-PMO shows a signal at 162.98 ppm corresponding to the carbonyl group of the amide side chain. Moreover, the signals arising from the pyridine ring are clearly visible at 143.00, 134.16, and 127.36 ppm [22]. Additionally, several signals at a higher field can be assigned to the resonances of the propylene group at 62.22 ppm (N–CH_2_), 23.42 ppm (–CH_2_–), and 7.04 ppm (Si–CH_2_) in the PMO-Py chain.

**Figure 4:**
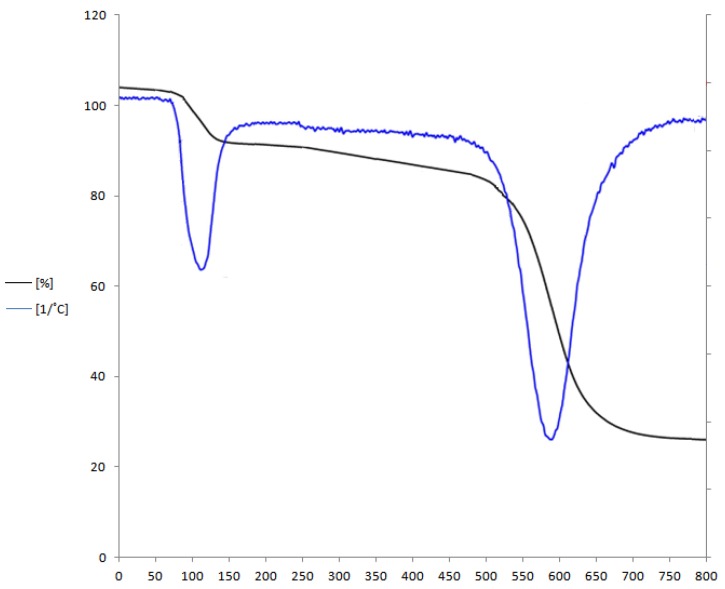
Thermogravimetric analysis/differential thermogravimetric (TGA/DTG) curves corresponding to the PMO-Py material up to 800 °C.

The characterization performed using thermogravimetric analysis/differential thermogravimetric analysis (TGA/DTGA) was employed to assess the thermal stability of PMO nanomaterials bearing pyridinedicarboxamide. Such an analysis was conducted from room temperature to 800 °C with a heating rate of 10 °C/min under static air atmosphere, and the respective TG and DTG curves of the PMO-Py are displayed in Figure 4. Firstly, a weight loss of approximately 9.7% owing to physisorbed water, ethanol, and loss of water by the condensation of residual silanol and ethoxy groups up can be observed up to 150 °C. The following important weight loss (ca. 60.5%) taking place between 500 and 650 °C, as particularly visible in the DTG curve, is ascribed to the complete collapse of structure and thermal decomposition of the organic moieties. Therefore, it could be concluded that as-synthesised PMO-Py materials show a rather stable behavior towards temperature, keeping the framework of the PMO-Py material stable up to 500 °C. Thus, further modifications could be carried out without a significant degradation of the mesoporous structure.

**Figure 5:**
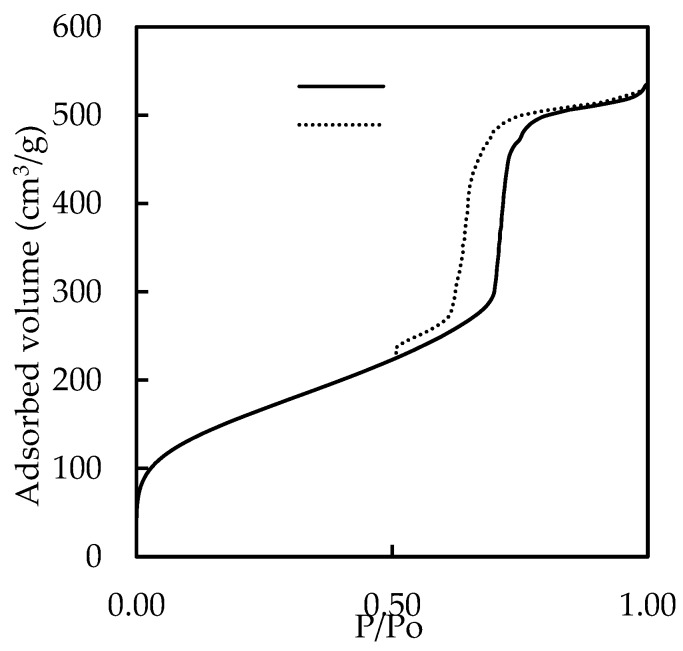
N_2_ adsorption/desorption isotherm plots for the Py-PMO material obtained at the liquid nitrogen temperature.

Nitrogen adsorption/desorption isotherm plots of the PMO-Py material (Figure 5) pointed to type IV isotherm curves, according to the IUPAC classification, characteristic of mesoporous materials. The synthesized materials displayed a high surface area of 616 m^2^/g, as determined by the Brunauer–Emmet–Teller (BET) equation. In addition, the pore size distribution curve reveals a very narrow pore distribution centered at 7.5 nm (as can be seen in Figure S1) in the mesopore range, and confirms the ordered structure of the Py-PMO material. Finally, it is important to also highlight the high pore volume of this material 0.68 cm^3^/g, calculated according to the Barrett, Joyner, and Halenda (BJH) method.

**Figure 6:**
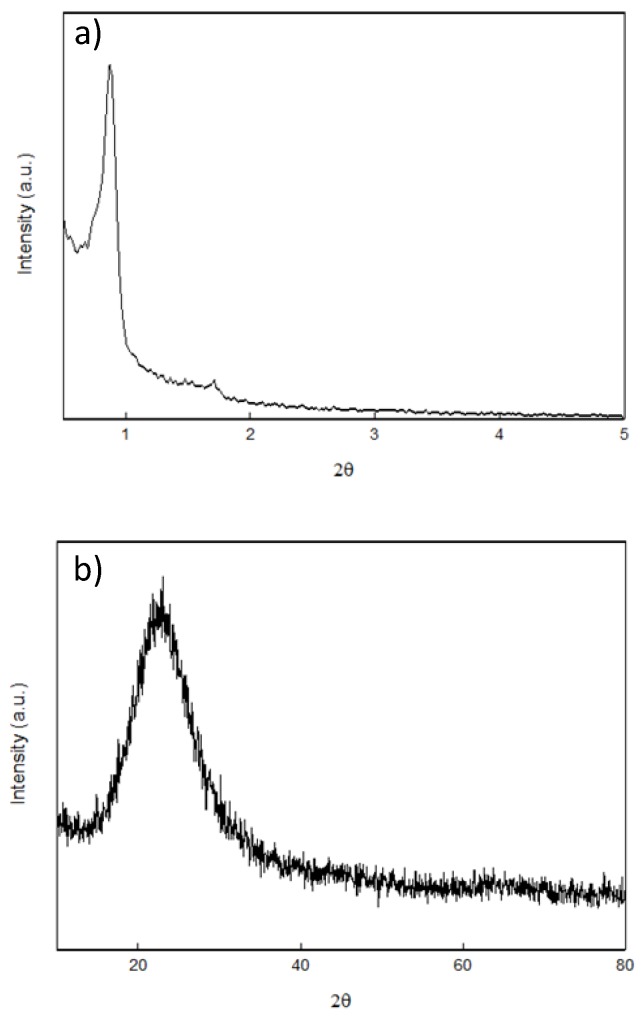
X-ray diffractograms corresponding to the PMO-Py material: (**a**) low-angle, (**b**) wide-angle.

Regarding the structure of as-synthesised PMO-Py material, the low angle X-ray diffraction (XRD) pattern (Figure 6a) clearly reveals three signals; the first is a sharp signal corresponding to the (100) reflection and two other less intense signals assigned to the (110) and (200) second-order reflections are clearly visible. Thus, the PMO-Py material presents well ordered 2D-hexagonal corresponding to the *P*6*mm* space group. In addition, wide-angle X-ray diffractograms (Figure 6b) did not show any crystal phase revealing an amorphous nature of the synthesized material.

In this material, in the presence of 2,6-diamide substitution in the pyridine ring, most of the pyridine units in the PMO structure (PMO-Py) are much less basic as compared with pyridine. In contrast, this substituted derivative of pyridine makes strong ligands in forming complexes with transition metal ion because of the chelate effect. Thus, this basicity in the PMO-Py material makes it a suitable catalyst for the Knoevenagel condensation reaction, which is one of the most important reactions and is very useful, and one that has been widely employed for the formation of carbon–carbon bonds in organic synthesis [23,24,25]. These condensation reactions occur between carbonyl compounds, either aldehydes or ketones, and compounds containing methylene activated groups. This reaction is widely employed as a carbon–carbon bond coupling reaction in the preparation of important intermediates in drug production by the pharmaceutical industry [26]. Therefore, the Knoevenagel reaction has been traditionally used for the evaluation of the catalytic activity of solid base catalysts including PMO materials [25]. 

**Scheme 2:**
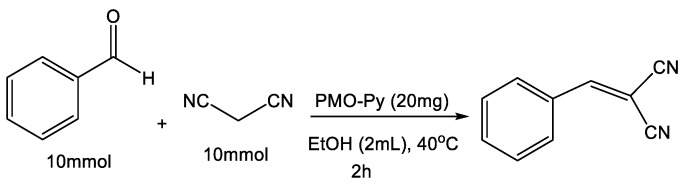
Knoevenagel condensation between malononitrile and benzaldehyde derivatives catalyzed by PMO-Py.

The catalytic activity of PMO-Py was subsequently evaluated in the Knoevenagel condensation of aromatic aldehydes with an active methylene compound such as malononitrile. In this sense, the reaction conditions were optimized for 10 mmol of malononitrile and 10 mmol of benzaldehyde (also substituted compounds) using 20 mg of PMO-Py as a catalyst using ethanol as solvent, according to Scheme 2.

Firstly, it is important to note that the PMO-Py material showed high conversion values for a relatively short reaction time under the investigated experimental conditions. There was not any additional product detected, so the selectivity was complete towards the C–C coupling product. In addition, the catalytic material developed in this study was also investigated in the Knoevenagel reaction between other benzaldehyde derivatives, with malononitrile obtaining excelling catalytic activity results for these substrates as well. The differences found in the conversion for the different benzaldehyde derivatives were because of their electronic as well as steric effects (Table 1). As It has been previously reported [27,28], the yield should be increased by the presence of electron withdrawing groups such as –Cl and –NO_2_. Nevertheless, this behavior it is not clear when PMO-Py is used as a catalyst after two hours, when the reaction is almost complete independently of the benzaldehyde used for the condensation with malononitrile. On the other side, when the substituents are electron releasing groups (–OCH_3_, –CH_3_), the conversion is slightly reduced as compared with the reaction using the non-substituted benzaldehyde. In addition, the effect of the use of different stereoisomers substituted benzaldehydes was investigated. In all of the cases, the use of the 4- regiomer (para-) led to the higher conversion as compared with the respective isomer substituted in 3- or 2- positions (ortho- or meta-) owing to the steric hindrance effect.

**Figure 7:**
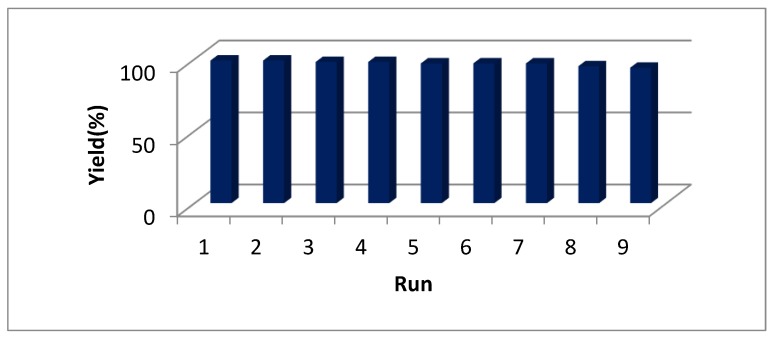
Reusability of the PMO-Py material in Knoevenagel condensation reaction between benzaldehyde and malononitrile under optimized conditions.

PMO-Py stability and recyclability in the Knoevenagel condensation reaction were also investigated. With this aim, the catalyst was isolated from the reaction mixture by filtration, washed with ethanol, and dried after each run for the following use in the Knoevenagel reaction. Thus, PMO-Py material exhibited a high stability without showing any significant catalytic activity loss after the 10th run in the condensation reaction between benzaldehyde and malononitrile under optimized conditions (Figure 7).

Table 2 show a comparison of the effectiveness of PMO-Py, with others reported in literature for the Knoevenagel reaction of benzaldehyde and malononitrile. The material herein synthesized according to a simple approach offers enhanced catalytic activity as compared with other basic catalytic materials investigated in the Knoevenagel reaction such as chitosan, a polysaccharide constitute of glucosamine units, which achieved analogous conversion as the PMO-Py material developed in this work after 4 h reaction, twice as high as the time required by the PMO-Py catalyst [28]. In addition, the conversion level achieved by the PMO-Py material improves the results achieved by other materials such as amino-functionalized mesoporous silicas [27], and is not far from others such as MgO/ZrO [29].

In addition to these, there are also relevant metal-encapsulated systems able to provide interesting results in knoevenagel chemistries as well as in biomedicinal applications [33,34].

## 3. Experimental

### 3.1. Synthesis of Diethyl Pyridine-2,6-Dicarboxylate

Diethyl pyridine-2,6-dicarboxylate, employed as a bridging group, was prepared in a 250 mL round-bottom flask equipped with a magnetic stirring bar, which was loaded with 50 mmol of pyridine-2,6-dicarboxylic acid and 1 mmol of p-toluenesulfonic acid (p-TsOH) in 100 mL ethanol. Then, the reaction mixture was stirred and refluxed for 2 h and, eventually, upon reaction completion (followed by thin layer chromatography (TLC)), the solvent was removed in a rotary evaporator, obtaining a solid residue. The diethyl pyridine-2,6-dicarboxylate was obtained after dissolving the solid residue in about 30 mL of diethyl ether (Et_2_O), which was subsequently washed with 3–5 portions of 5% NaHCO_3_ and water. Finally, the organic phase was dried over MgSO_4_, filtered, and the solvent was removed to obtain pure diethyl pyridine-2,6-dicarboxylate as a white solid with a 95% yield.

### 3.2. Synthesis of Bis(3-(Triethoxysilyl)Propyl)Pyridine-2,6-Dicarboxamide

A mixture of sodium ethoxide (0.1 mmol), diethyl pyridine-2,6-dicarboxylate (10 mmol), and 3-(triethoxysilyl)propyl amine (20.5 mmol,) was heated together in a sealed tube at 170 °C under stirring under nitrogen atmosphere for 5 h. After cooling down the reaction mixture, chloroform was poured into the reaction tube to extract the reaction product. Subsequently, the suspension was filtered and the solvent was removed under vacuum, obtaining pure *bis(3-(triethoxysilyl)propyl)pyridine-2,6-dicarboxamide* as a white solid in 92% yield. C_34_H_67_N_3_O_11_Si_3_ (778.17): the calculated composition was C: 52.48, H: 8.68, N: 5.40, while the actual one was C: 53.01, H: 8.24, N: 5.14.

### 3.3. Synthesis of PMO Materials Bearing Pyridinedicarboxamide (PMO-Py)

In a typical synthesis, 0.4 g of Pluronic P123 (Aldrich, average Mw ≅ 5800) was dissolved in 12.5 g of 2 M HCl solution with stirring at 35 °C. Then, 2.3 g of bis(3-triethoxysilyl)propyl)pyridine-2,6-dicarboxamide was added and the resulting mixture solution was vigorously stirred at 35 °C for 20 h. The mixture was aged at 90 °C overnight without stirring and, subsequently, the solid was filtered off and dried at 50 °C overnight. The template was removed from the material through solvent extraction: 1.0 g of the as-synthesized sample was refluxed in a solution of 1.0 mL 37% HCl and 100 mL ethanol for 12 h, and then washed thoroughly with a mixture ethanol/water using a Soxhlet apparatus for 24 h. Finally, it was dried in an oven at 80 °C overnight.

### 3.4. Material Characterisation

Solid-state ^13^C-NMR cross-polarization (CP) and magic angle spinning (MAS) spectra were recorded in a Bruker 300 MHz Ultrashield spectrometer (Rheinstetten, Germany). The sample spin rate was 8 kHz, proton length pulse was 2.5 µs, contact time was 2.4 ms, and repetition time was 3 s. 

The textural properties of the synthesized material were measured by nitrogen physisorption measurements carried out at 77 K using a Micromeritics ASAP 2000 instrument (Micromeritics, Norcross, GA, USA). Samples were outgassed for 24 h at 100 °C under vacuum (10^−4^ mbar), and subsequently analyzed. Surface area was calculated using the BET (Brunauer–Emmet–Teller) equation, while the pore volume (V_BJH_) and pore size distributions (D_BJH_) were obtained the BJH method (Barrett, Joyner, and Halenda) in the N_2_ adsorption branch.

Powder X-ray diffraction patterns were recorded on a Bruker-AXS diffractometer using Cu Kα radiation (λ = 1.5409 Å) (Rheinstetten, Germany). Wide-angle diffraction pattern was recorded in the range 10° ≤ 2*θ* ≤ 80°, with a step size of 0.017 and a scan time of 1.3 s. Low-angle X-ray diffractogram was acquired in the range 0.5° ≤ 2*θ* ≤ 5° with 5 s time per step and a step size of 0.01°.

Thermogravimetric analysis (TGA) was performed from 25 to 800 °C using a heating rate of 10 °C·min^−1^ under static air atmosphere by a NETZSCH STA 409 PC/PG Instrument (Selb, Germany).

### 3.5. Knoevenagel Reaction

The catalytic tests of the as-synthesised material were performed in a 50 mL round-bottomed flask equipped with a magnetic stirring bar, which was charged with 10 mmol aromatic aldehyde, 20 mg of catalyst (PMO-Py), and 10 mmol of malononitrile in 2mL EtOH. The reaction mixture was stirred at 40 °C for 2 h. After that, the reaction mixture was filtered and the solid was washed with ethanol. The reaction was followed by withdrawing samples periodically that were subsequently analyzed by gas chromatography using an Agilent 6890N GC model equipped with a fitted with a DB-5 capillary column and an FID detector. To study the reusability of PMO-Py, the catalyst was separated after each cycle from the reaction mixture by simple filtration, washed with hot ethanol and dried at vacuum oven at 60 °C, and employed again in another reaction run.

## 4. Conclusions

A general and simple protocol was developed to synthesize periodic mesoporous organosilicas (PMOs) materials with bisilylated bridged pyridinedicarboxamide moieties uniformly distributed in the nanostructure pore walls. As-synthesized catalysts displayed a mesoporous nature with an arrangement typical for hexagonal ordered materials. The incorporation of pyridinedicarboxamide functionalities provided the as-prepared materials with the basic nature that was evaluated in the Knoevenagel condensation reaction.

The functionalized mesoporous organosilica proved to be a highly active, stable, and recyclable heterogeneous basic catalyst for the Knoevenagel condensation reactions between benzaldehyde and derivatives with malononitrile. Such pyridinedicarboxamide functionalized mesoporous organosilicas are expected to serve as a coordinated ligand to transition metals for further post-modifications and catalytic applications. Further studies on the preparation of catalysts containing transition metal are presently being carried out in our group.

## Figures and Tables

**Table 1:** Knoevenagel condensation reaction of various aldehydes with malonitrile catalyzed by the periodic mesoporous organosilica (PMO)-Py catalyst.

Entry	R	Conversion (%)	Selectivity (%)
1	H	99	>99
2	4-NO_2_	98	>99
3	3-NO_2_	95	>99
4	4-Cl	99	>99
5	2-Cl	95	>99
6	4-OMe	94	>99
8	2-Me	92	>99
9	4-Me	90	>99
10	Blank	-	-

**Table 2:** Comparison of the catalytic efficiency of PMO-Py in the reaction of benzaldehyde, malononirile and the previous literature.

Entry	Catalyst	Condition	Time	Conversion (%)	Ref.
1	PMO-Py (20 mg)	PMO-Py	2 h	99	This work
2	amino-functionalized mesoporous silica (20 mg)	EtOH/RT	6 h	90	[27]
3	Chitosan (25 mg)	EtOH/40 °C	6 h	99	[28]
4	MgO/ZrO_2_ (20 wt%)	60 °C	20 min	93	[29]
5	PMO-IL-NTf2 (0.2 g)	EtOH/RT	20 min	96	[30]
6	SO_3_H-AA@MNP (20 mg)	H_2_O-toluene/90 °C	2 h	94	[31]
7	Chol-SBA-15 (5mg)	EtOH/60 °C	60 min	100	[32]

## Supplementary Materials

The following are available online at https://www.mdpi.com/1996-1944/13/5/1097/s1, Figure S1: Pore size distribution curve corresponding to the material PMO-Py.
