# Supplementary material for: Synthesis and Characterization of Novel Pyridine Periodic Mesoporous Organosilicas and Its Catalytic Activity in the Knoevenagel Condensation Reaction
Source: Materials (Basel). 2020 Mar 2;13(5):1097. doi: 10.3390/ma13051097 (PMC7084680; doi:10.3390/ma13051097)
Supplement: Supplementary file 1 [file materials-13-01097-s001.zip › materials-712795-SI.docx]

Article

**ESI**

Synthesis and Characterization of Novel Pyridine Periodic Mesoporous Organosilicas and Its Catalytic Activity in the Knoevenagel Condensation Reaction

Fatemeh Rajabi ^1,^*, Arezoo Zare Ebrahimi ^1^, Ahmad Rabiee ^2^, Antonio Pineda ^3^, and Rafael Luque ^3^

^1^ Department of Science, Payame Noor University, P. O. Box: 19395-4697, Tehran 19569, Iran; arezou.ebrahimi68@gmail.com

^2^ Iran Polymer and Petrochemical Institute (IPPI), P.O. Box 112/14975, Tehran 19569, Iran; a.rabbii@ippi.ac.ir

^3^ Department of Organic Chemistry, University of Cordoba, Ed. Marie Curie (C 3), Campus of Rabanales, Ctra Nnal IV-A, Km 396, E14014 Cordoba, Spain; q82pipia@uco.es (A.P.); q62alsor@uco.es (R.L.)

***** Correspondence: f_rajabi@pnu.ac.ir (F.R.)

Received: 20 January 2020; 18 February 2020; Published: date

Spectroscopic data of Knoevenagel reaction

*2-Benzylidenemalononitrile*: m.p. 83-84^o^C; ^1^H NMR (400 MHz, CDCl_3_): δ7.54 (2 H, t, *J* = 8.0 Hz), 7.64 (1 H, t, *J* = 7.6 Hz), 7.79 (1 H, s), 7.92 (2 H, d, *J* = 7.6 Hz) ppm. ^13^CNMR (100 MHz, CDCl_3_): δ 82.7, 112.5, 113.7, 129.5, 130.7, 131.0, 134.6, 159.9ppm.

*2-(4-Nitrobenzylidene)malononitrile*: ^1^H NMR (400 MHz, CDCl_3_): m.p. 158-159 ºC δ 7.82 (1 H, s), 8.06 (2 H, d, *J* = 8.7 Hz), 8.38(2 H, d, *J* = 8.7 Hz)ppm.^13^CNMR (100 MHz, CDCl_3_): δ 87.4, 111.5, 112.5, 131.2, 135.5, 149.9, 156.8 ppm.

*2-(3-Nitrobenzylidene)malononitrile*: ^1^H NMR (400 MHz, CDCl_3_): m.p. 99-100 ºC; δ 7.85 (1 H, s), 8.30 (1 H, s ), 8.39(m, 3H) ppm. ^13^CNMR (100 MHz, CDCl_3_): δ 85.4, 113.1, 113.9, 125.0, 127.9, 131.2, 132.5, 135.8, 148.9, 158.8 ppm.

*2-(4-Chlorobenzylidene)malononitrile*: m.p. 160-161 ºC; ^1^H NMR (400 MHz, CDCl_3_): δ 7.52 (2 H, d, *J* = 8.5 Hz), 7.73 (1 H, s), 7.85 (2 H, d, *J* = 8.5 Hz)ppm. ^13^CNMR (100 MHz, CDCl_3_): δ 83.1, 112.3, 113.5, 129.2, 129.9, 131.8, 141.1, 158.4ppm.

*2-(2-Chlorobenzylidene)malononitrile*: m.p. 78-80 ºC; ^1^H NMR (400 MHz, CDCl_3_): δ 7.70 (2H, m), 7.75 (1 H, s), 7.81 (2 H, m)ppm. ^13^CNMR (100 MHz, CDCl_3_): δ 83.5, 112.4, 113.5, 129.7, 129.9, 131.6, 133.0, 157.9 ppm.

*2-(4-Methoxybenzylidene)malononitrile*: m.p. 115-116 ºC; ^1^H NMR (400 MHz, CDCl_3_): δ 3.91(3H, s), 6.97(2 H, d, *J* = 8.1 Hz), 7.67 (1H, s), 7.93(2 H, d, *J* = 8.1 Hz)ppm. ^13^CNMR (100 MHz, CDCl_3_): δ 55.7, 78.5, 113.3, 114.4, 115.1, 124.0, 130.5, 158.8, 164.8ppm.

*2-(4-Methylbenzylidene)malononitrile*: m.p. 135-136ºC; ^1^H NMR (400 MHz, CDCl_3_): δ 3.06(3H, s), 7.24(2 H, d, *J* = 8.2 Hz), 7.40 (1H, s), 7.63(2 H, d, *J* = 8.2 Hz)ppm. ^13^CNMR (100 MHz, CDCl_3_): δ 22.2, 81.3, 113.0, 114.1, 128.6, 130.5, 131.1, 145.6, 159.9 ppm.

*2-(2-Methylbenzylidene)malononitrile*: m.p. 102-104 ºC; ^1^H NMR (400 MHz, CDCl_3_): δ 8.47 (1H, s), 8.02 (1 H, d, *J*= 7.9Hz), 7.51(1 H, m), 7.35 (2H, m), 2.43 (3H, s)ppm.^13^CNMR (100 MHz, CDCl_3_): δ 19.7, 85.3,113.9, 114.8, 127.5, 129.1, 131.5, 132.6, 134.6, 141.0, 159.8 ppm.


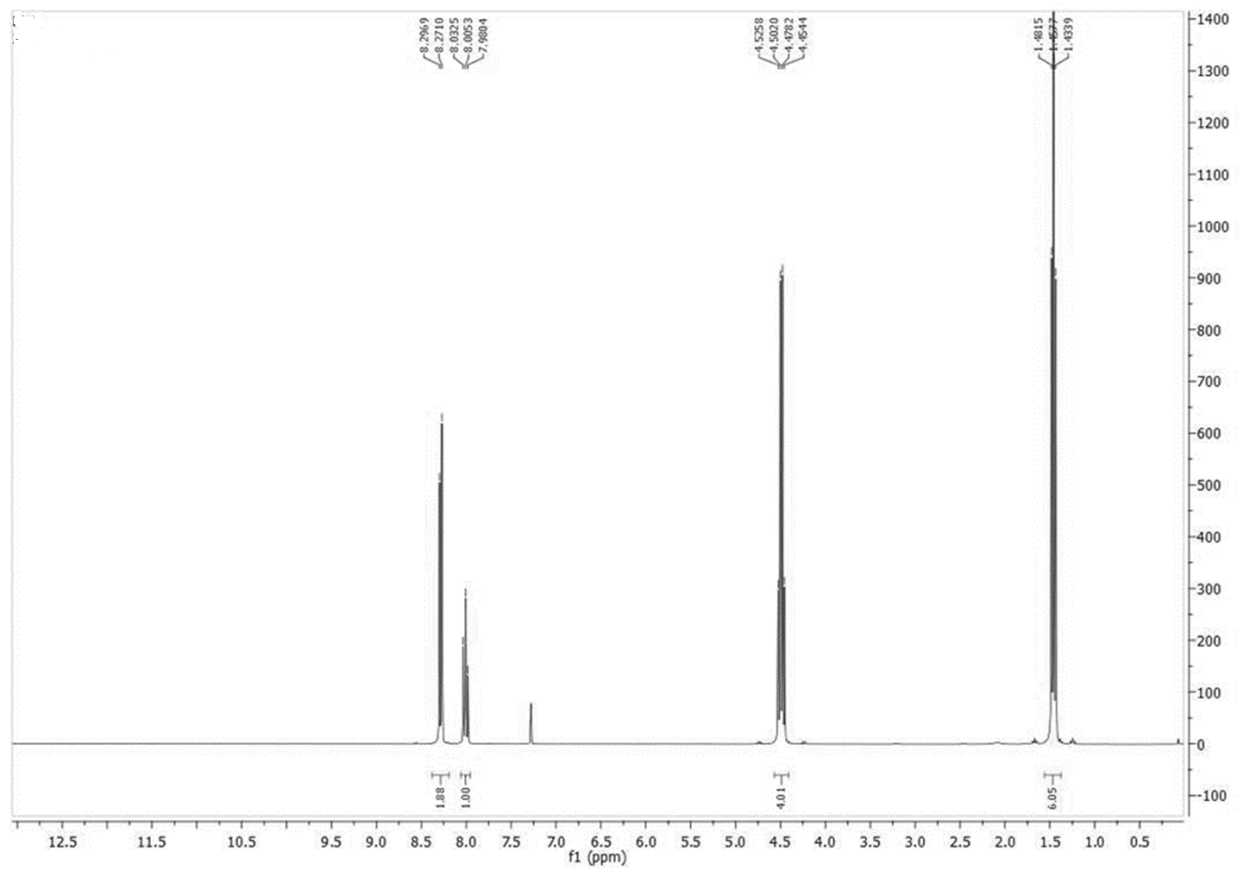


**Figure S1.** ^1^HNMR spectrum diethyl pyridine-2,6-dicarboxylate.

**
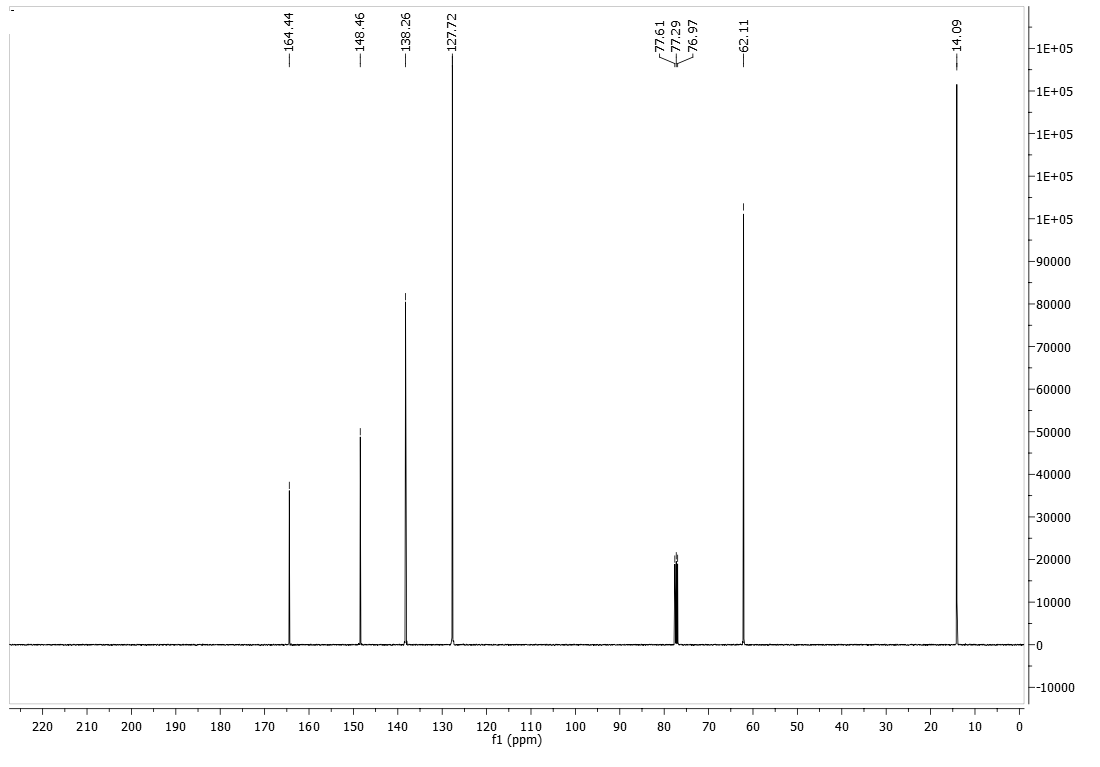
**

**Figure S2.** ^1^HNMR spectrum diethyl pyridine-2,6-dicarboxylate.


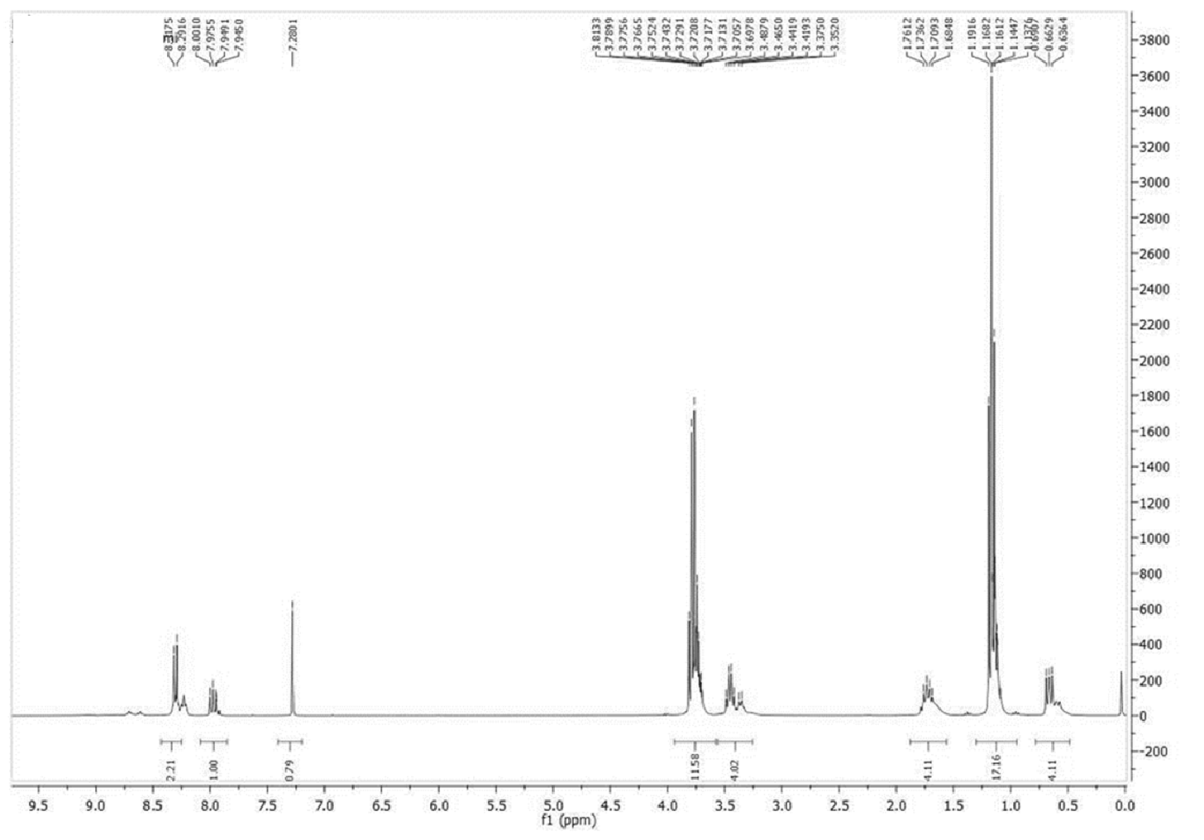


**Figure S3.** ^1^HNMR spectrum bis(3-(triethoxysilyl)propyl)pyridine-2,6-dicarboxamide.

**
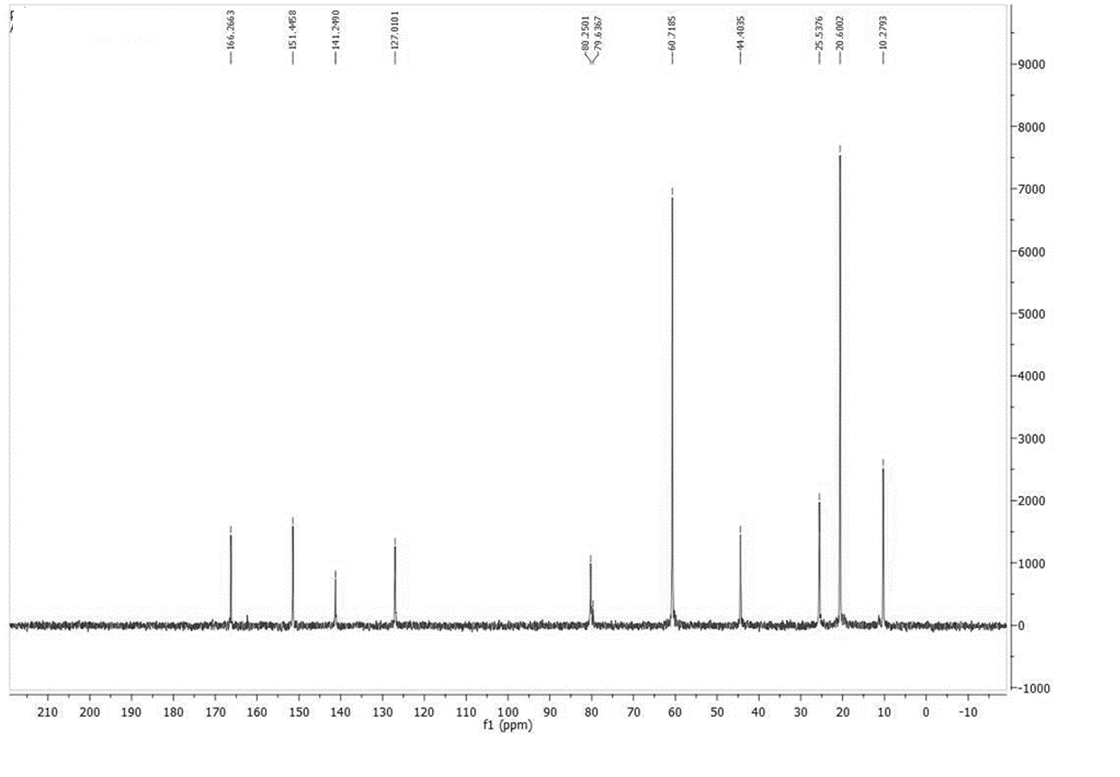
Figure S4.** ^13^CNMR spectrum bis(3-(triethoxysilyl)propyl)pyridine-2,6-dicarboxamide.

**Figure S5.** Pore size distribution curve corresponding to the material PMO-Py.

| 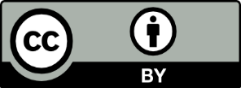 | © 2020 by the authors. Submitted for possible open access publication under the terms and conditions of the Creative Commons Attribution (CC BY) license (http://creativecommons.org/licenses/by/4.0/). |
| --- | --- |
